# Improved symptomatic, functional, and fluoroscopic outcomes following serial “series of three” double-balloon dilation for cricopharyngeus muscle dysfunction

**DOI:** 10.1186/s40463-018-0278-7

**Published:** 2018-05-15

**Authors:** Derrick R. Randall, Lisa M. Evangelista, Maggie A. Kuhn, Peter C. Belafsky

**Affiliations:** 1 Center for Voice and Swallowing, Department of Otolaryngology – Head & Neck Surgery, University of California Davis, Sacramento, CA USA; 2 Section of Otolaryngology – Head & Neck Surgery, Department of Surgery, University of Calgary, Calgary, AB T2W 3K2 Canada

**Keywords:** Dysphagia, Cricopharyngeus muscle dysfunction, Otolaryngology, Laryngology, Swallowing, Transnasal esophagoscopy

## Abstract

**Background:**

Cricopharyngeus muscle dysfunction (CPMD) is a common cause of dysphagia. We employ a progressive series of three double-balloon dilations separated by 4–6 weeks between procedures as a primary treatment option. The purpose of this study was to evaluate subjective, functional and objective improvement in swallowing after three serial dilations for CPMD.

**Methods:**

We retrospectively evaluated patients between June 1, 2014, and June 30, 2016, who underwent a series of three double-balloon dilations for CPMD. Pre- and post-dilation Eating Assessment Tool-10 (EAT-10), Functional Oral Intake Scale (FOIS), pharyngeal constriction ratio, pharyngeal area, and pharyngoesophageal segment (PES) opening were compared.

**Results:**

Seventeen patients with CPMD underwent serial double-balloon dilation procedures separated by one month. Mean age of the cohort was 73.5 (SD ± 13.3) years, and 53% were female. The mean EAT-10 improved from 24.7 (SD ± 7.8) to 15.9 (SD ± 10.2) [*p* = 0.0021]. Mean FOIS improved from 5.4 (SD ± 1.4) pre- to 6.3 (SD ± 0.9) post-treatment (*p* = 0.017). Mean UES opening increased from 1.05 (SD ± 0.34) cm to 1.48 (SD ± 0.41) cm (*p* = 0.0003) in the anteroposterior fluoroscopic view and from 0.58 (SD ± 0.18) to 0.76 (SD ± 0.30) cm (*p* = 0.018) in the lateral view. Pharyngeal constriction ratio (PCR), a surrogate measure of pharyngeal strength, improved from 0.49 (SD ± 0.37) to 0.24 (SD ± 0.15) (*p* = 0.015), however pharyngeal area (PA) was unchanged.

**Conclusions:**

A progressive series of three double-balloon dilations for cricopharyngeus muscle dysfunction resulted in improved patient reported dysphagia symptom scores and objective fluoroscopic swallowing parameters.

## Background

Oropharyngeal swallowing dysfunction is common and costly. Complications include malnutrition, dehydration, depression, social isolation, pneumonia, hospital admission, increased length of stay, and death [1–3]. Early recognition allows implementation of appropriate rehabilitation, diet allocation or surgical management to prevent sequelae of impairment [4–6]. The pharyngoesophageal segment (PES) is a manometric high-pressure zone extending 3–5 cm from the hypopharynx to the cervical esophagus. Dysphagia resulting from PES dysfunction is a result of obstruction, poor compliance, impaired laryngeal elevation or ineffective pharyngeal propulsive forces [7]. Persons with PES dysfunction may present with solid food dysphagia, choking with deglutition, throat clearing, and globus.

**Fig. 1:**
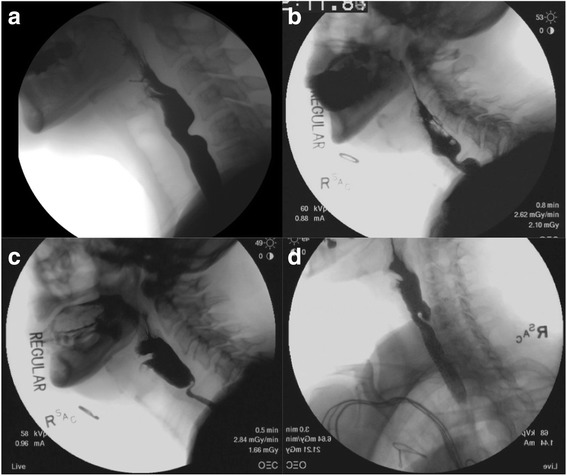
Spectrum of cricopharyngeus muscle dysfunction showing asymptomatic narrowing of pharyngoesphageal segment to severe narrowing and diverticulum formation. **a** Non-obstructing bar. **b** Moderately obstructing bar. **c** Severely obstructing bar. **d** Zenker’s diverticulum

One of the most common causes of solid food oropharyngeal dysphagia is cricopharyngeus muscle dysfunction (CPMD) [8]. The cricopharyngeus muscle is an essential component of the PES and is responsible for preventing the ingestion of air during respiration and reflux of esophageal contents into the pharynx. CPMD manifests as a spectrum of videofluoroscopic swallowing study (VFSS) findings ranging from non-obstructing bars found in up to 30% of the asymptomatic population to severely obstructing bars that limit oral intake to solids and liquids (Fig. 1) [9]. Pharyngeal constriction against an obstructed PES can lead to the development of a dilated, weak pharynx and Zenker’s diverticulum [10, 11]. Treatment options include diet modification, botulinum toxin injection, dilation and endoscopic or open myotomy [7]. A variety of procedural interventions have been demonstrated to improve both symptoms and radiographic evidence of CPMD [7, 12, 13]. The optimal treatment requires an individualized strategy that takes into account disease severity, patient comorbidities and functional status, prognosis and required duration of effect.

A recent systematic review of dilation for CPMD found a variable response rate, ranging from 64 to 100% [12]. The variability in treatment efficacy may be related to dilator size, number of procedures, and the underlying disease process [14–17]. Furthermore, measures of swallowing function can be assessed by one of the numerous dysphagia symptom indices, if at all, or objective outcomes from fluoroscopic investigations. In our center, we use a series of progressively enlarging balloon dilations, utilizing two balloons simultaneously to achieve a greater dilation profile and better approximate the natural, kidney shape of the PES [18, 19]. Patients are asked to complete Eating Assessment Tool (EAT-10) and Functional Oral Intake Scale (FOIS) instruments at all patient encounters in order to measure their progress and assess the severity of their symptoms. The EAT-10 has been validated for impact on quality of life due to dysphagia for several different etiologies, while the FOIS evaluates the degree of oral diet capacity by considering variety of consistencies a patient can manage and the amount of enteral tube feeding required [20, 21]. The purpose of this investigation was to determine the short term subjective and objective outcomes of serial PES double-balloon dilation for CPMD.

## Methods

### Patient population and outcome measures

**Fig. 2:**
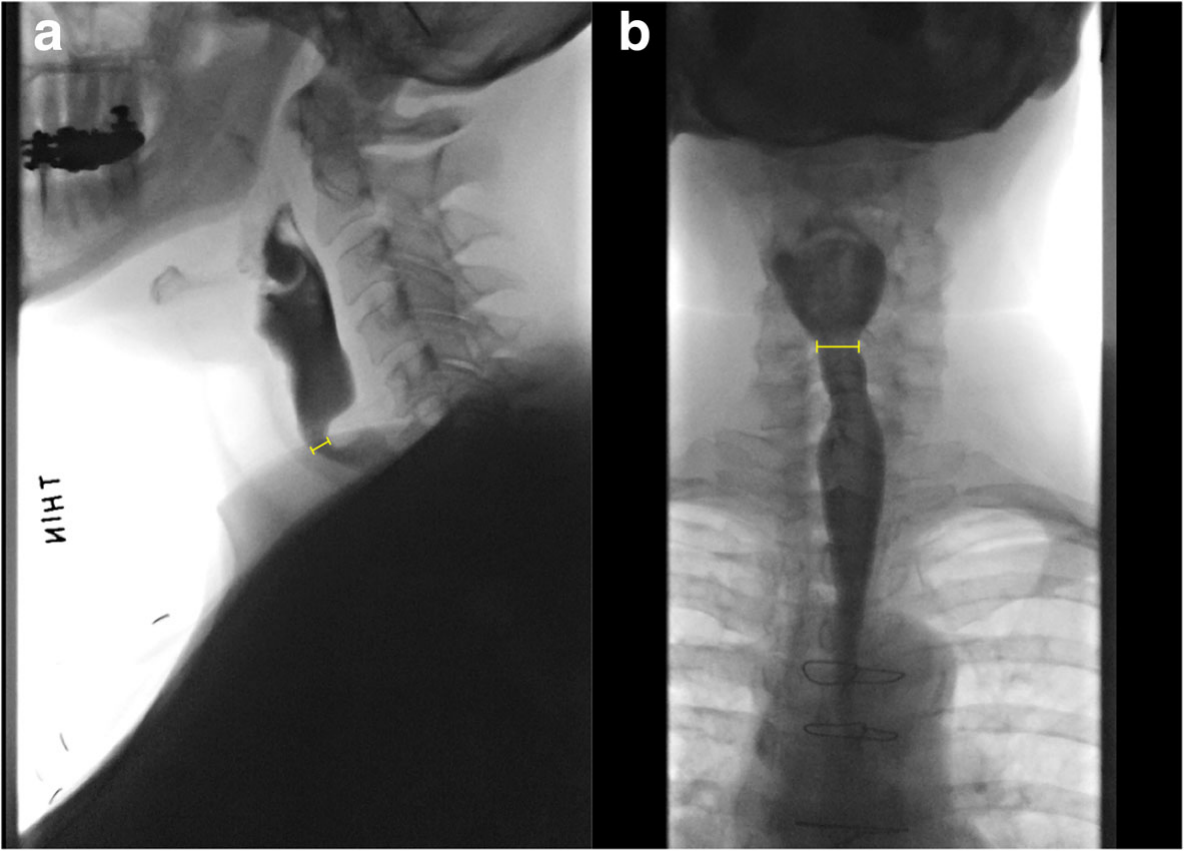
Demonstration of pharyngoesophageal segment (PES) parameters in videofluoroscopy studies. **a** PES lateral view (PESL). **b** PES anteroposterior view (PESAP). Measurement bars indicate narrowest opening dimension during 20 mL barium volume test

This investigation was approved by the University of California, Davis Institutional Review Board (protocol #905351–1). All patients with complete data who underwent a series of three progressively increased balloon dilations for CPMD between June 1, 2014, and June 30, 2016 were included. The diagnosis of CPMD was made on VFSS. Patients who had undergone intervention for CPMD prior to the VFSS and those with either Zenker diverticulum or prior radiation therapy of the head and neck were excluded. Pre- and post-dilation validated EAT-10, FOIS, PES opening (cm) in the anteroposterior (PESAP) and lateral (PESL) fluoroscopic view (Fig. 2), pharyngeal constriction ratio (PCR), and pharyngeal area (PA) were retrospectively collected.

### Videofluoroscopic analysis

Fluoroscopic studies were performed according to our center’s standard protocol [22, 23]. Each subject was administered a bolus of liquid barium (EZ-PAQUE barium sulfate suspension, 60% *w*/*v*; 41% *w*/w, E-Z-EM, Inc., Westbury, NY) in the following order: 1, 3, and 20 mL. Each subject was also given a 3-cm^3^ bolus of barium paste (EZpaste, E-Z-Em, Inc). Patients undergoing esophagram did not receive a 3 mL bolus of liquid or paste barium but did additionally receive a 13 mm barium tablet and large volume (> 60 mL) liquid barium trial via straw drinking. The fluoroscopic studies were recorded digitally with Olympus Image Stream Medical nStream G3 HD/SD Video Recording (Image Stream Medical, Littleton, MA) and were played back with WinDVD7 for Windows (Intervideo, Corel Corp., Ottawa, Canada).

Objective fluoroscopic displacement measures were obtained according to established protocols [9, 24–26]. In brief, pharyngeal area is measured in the lateral view on the 1 mL liquid barium ‘hold’ position. The posterior landmark starts superiorly at the posterior pharyngeal wall anterior to the tubercle of the atlas and follows inferiorly to the floor of the hypopharynx. The anterior boundary is traced from the posterior arytenoids to the surface of the arytenoid cartilages, proceeds to the laryngeal surface of the epiglottis, curves into the valleculae, then follows along the base of tongue. The anterior/superior landmark ends at the velum. All measures requiring lateral views utilize these landmarks. PCR is the ratio of PA at maximal compression divided by the PA at rest and is a validated measure of pharyngeal contractility. An elevated PCR suggests diminished pharyngeal strength [23]. Standardized measures of the PESL and PESAP were obtained during the trial of 20 mL liquid barium bolus. Anteroposterior (AP) measures for the UES opening are obtained in the AP view with the same superior and inferior landmarks; lateral boundaries are defined as the maximal distention.

### Surgical technique

**Fig. 3:**
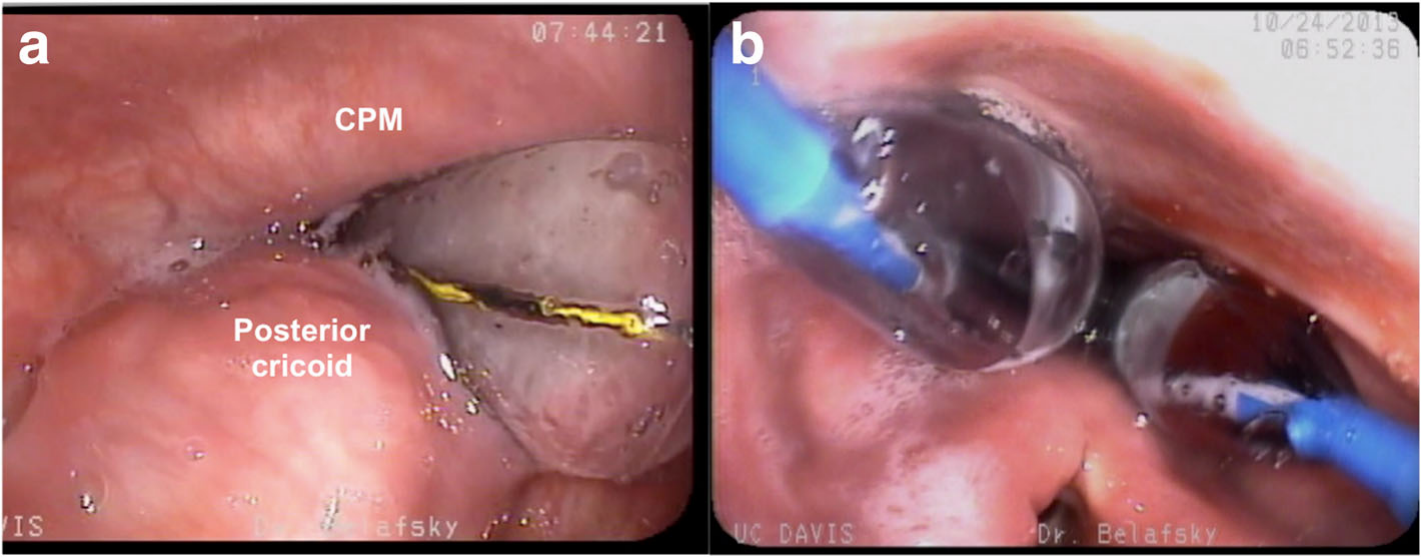
Transnasal esophagoscope view of pharynx during sequential balloon dilation using **a**) one and **b**) two balloon dilators. CPM = cricopharyngeus muscle

All dilation procedures were performed under monitored anesthesia care with sedation administered per anesthesiologist preference. Typical sedation is achieved with a combination of midazolam and fentanyl. Our technique of dilation begins with the administration of a combination xylocaine (4%) and neosynephrine (0.25%) nasal spray administered 2–3 min prior to the procedure. Flexible esophagoscopy is performed through the more patent naris (Pentax VE-1530 transnasal esophagoscope, Pentax Precision Medical Company, KayPentax, Lincoln Park, NJ, USA). A diagnostic esophagoscopy is performed and a guidewire(s) from a Hercules® 3 Stage Wire Guided Balloon (Cook Medical, Bloomington, IN) is inserted through the side channel of the endoscope. The esophagoscope is then removed over the guidewire(s), replaced through the contralateral naris, and positioned in the hypopharynx to visualize the postcricoid region. The dilation balloon(s) are then advanced over the guidewire(s). Our protocol involves sequential dilation personalized for each patient but typically begins with one 18–20 mm balloon in the first procedure, followed by two 13–15 mm balloons in the second dilation and two 15–18 mm balloons for the third dilation (Fig. 3). Balloons were sequentially inflated through each diameter and then held at final dilation for 60 s. After deflation and removal, the PES was examined for signs of injury to the mucosa. The mucosa is examined at every stage of dilation and the procedure is terminated if any blood is seen on the balloon or evidence of mucosal laceration is visualized. The interval between dilations was 4–8 weeks, dependent on patient and OR availability, in accordance with use in prior studies [27, 28].

### Statistical analysis

Statistical analysis was carried out using Stata 12.0 (StataCorp, College Way, TX), with descriptive statistics determined for baseline and demographic data. Comparison between pre- and post-dilation outcomes was performed using paired t-tests for continuous variables and Wilcoxon matched pairs–signed rank test for ordinal data. A Bonferroni correction was utilized to adjust for multiple comparisons.

## Results

Seventeen patients with CPMD who underwent three serial balloon dilations with complete pre- and post-treatment fluoroscopy data were enrolled. The mean age of the cohort was 73 (SD ± 11.5) years, 59% female. Fifty-one dilations were done in total. The mean duration between pre- and post-treatment VFSS was 206 (SD ± 83) days and the mean time between final dilation and post-treatment fluoroscopy was 37 (SD ± 33) days. Median balloon dilator diameters for the first, second, and third stages of the “series of three” dilations were 20 mm, 15 + 15 mm, and 18 + 18 mm, respectively, situated in the PES as shown in Fig. 3.

Changes in symptom and functional scores are displayed in Table 1. The mean EAT-10 improved from 24.7 (SD ± 7.8) pre- to 15.9 (SD ± 10.2) post-treatment (*p* = 0.0021). The mean FOIS improved from a mean of 5.4 (SD ± 1.4) pre- to 6.3 (SD ± 0.9) post-treatment (*p* = 0.017). In our series, individual patient EAT-10 and FOIS scores either improved or were unchanged in 14/17 patients (82%) following the third dilation, compared to pre-treatment values.

**Table 1:** Pre-and post-treatment symptom scale and functional outcome measures

Parameter	*n*	Pre-treatment (+/− SD)	Post-treatment (+/− SD)	*p* value
EAT-10	17	24.7 (7.8)	15.9 (10.2)	0.0021*
FOIS	17	5.4 (1.5)	6.3 (0.9)	0.017**

*EAT-10* eating assessment tool, *FOIS* functional oral intake scale

*Paired t-test

**Wilcoxon matched pairs–signed rank test

**Fig. 4:**
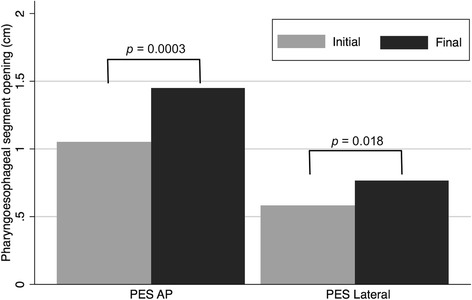
Mean values of pre- and post-dilation opening dimensions of the pharyngoesphageal segment (PES). AP = anteroposterior view

Changes in radiographic outcome are displayed in Table 2. PESAP increased from 1.05 cm (SD ± 0.34 cm) to 1.48 cm (SD ± 0.41 cm) (*p* = 0.0003). PESL increased from 0.58 cm (SD ± 1.8 cm) to 0.76 cm (SD ± 0.30 cm) (*p* = 0.018). These values represent increases in PES opening width and anteroposterior space of 41 and 31%, respectively (Fig. 4). Among patients with severely obstructed CPMD (PESL opening less than 0.5 cm, *n* = 7) the anteroposterior opening increased by 72% from 0.39 cm (SD ± 0.07 cm) to 0.67 cm (SD ± 0.27 cm) (*p* = 0.047). Patients who underwent serial balloon dilation also showed improvement in PCR from 0.49 (SD ± 0.37) to 0.23 (SD ± 0.15) (*p* = 0.015), indicating improved ability to constrict the pharynx and propel a food bolus through the PES. Despite improved constriction, PA showed no difference between pre- and post-treatment values (*p* = 0.91). There were no perforations of the upper esophagus or PES identified in this study cohort, and no patients developed delayed infections in the neck.

**Table 2:** Pre-and post-treatment VFSS outcome measures. All tests performed with paired t-tests

Parameter	*n*	Pre-treatment (+/− SD)	Post-treatment (+/− SD)	*p* value
PESAP opening (cm)	15	1.05 (0.34)	1.48 (0.41)	0.0003
PESL opening (cm)	17	0.58 (0.18)	0.76 (0.30)	0.018
Pharyngeal constriction ratio	13	0.49 (0.37)	0.24 (0.15)	0.015
Pharyngeal area (cm^2^)	12	9.45 (3.62)	9.52 (3.96)	0.91

*PESAP* upper esophageal sphincter in anterior-posterior view, *PESL* upper esophageal opening in lateral view

To evaluate the durability of our treatment approach, we reviewed patient records to determine whether members of our cohort underwent additional treatments. We identified ten patients (59%) who underwent additional upper esophageal procedures, nine of whom had repeat dilations and one who opted for a cricopharyngeus myotomy. The mean duration of time between the third dilation and a repeat procedure was 416 days (SD ± 246, range = 124–849 days). Five patients who underwent subsequent treatments also had fluoroscopic studies repeated, however there were no statistically significant differences between the measures obtained after the third dilation and the later dilation. Similarly, there were no differences between EAT-10 or FOIS scores (*n* = 9) following additional treatments.

## Discussion

In this study, we investigated the effect of serial PES dilation for CPMD and report improved patient-reported symptoms, functional, and fluoroscopic short-term outcomes. We observed marked improvements in PES opening and EAT-10 scores, confirming existing data that PES dilation is an effective method to address CPMD [12]. Though symptom scores do not return to normal after intervention, they suggest significant improvement, which is compatible with measured fluoroscopic outcomes.

Our study provides several interesting findings related to the efficacy of serial double-balloon dilation to a size larger than what can be achieved with a single balloon. As expected, both PESAP and PESL opening increase in these patients but the increase is more pronounced in the anterior posterior projection (PESAP). The fluoroscopic finding of a hypertrophic cricopharyngeus muscle used to diagnose CPMD is typically identified on the lateral VFSS projection. We did not expect the greatest therapeutic benefit to be appreciated in the anteriorposterior fluoroscopic view (PESAP). Previous fluoroscopic data report improvement in the lateral fluoroscopic view only (PESL) [10, 16]. Fig. 3 illustrates that the increased dilation obtained by using two balloons occurs in a vector that should increase the lateral dimension of the PES, a consequence that would result in improved opening on the AP VFSS. This degree of lateral expansion cannot be effectively achieved with a single, circular balloon or bougie.

Anectodal experience and case reports indicate reflux symptoms can worsen following cricopharyngeus myotomy in select populations [29–31], leading some authors to consider reflux or ineffective esophageal motility a contraindication [32]. However, manometric studies of PES pressures before and after cricopharyngeus muscle myotomy demonstrate reduction of resting pressure to normal values with no increase in pharyngeal acid regurgitation [33–35]. The cricopharyngeus muscle is not ablated or resected with dilation, and it has been reported that single balloon dilation reduces the size of an obstructing cricopharyngeus muscle less than myotomy [10]. We observed persistent fluoroscopic indentation of the PES in most patients post serial dilations. This suggests that serial dilation may cause less diminution to the protective function of the PES than myotomy.

Another interesting observation was the disparate change in PCR and PA following serial PES dilation. While pharyngeal contraction improved (PCR), the pharynx remained dilated (PA). These data are consistent with other reports after both dilation and myotomy [10], which suggests that some of the pharyngeal dilation caused by prolonged PES obstruction may be permanent. A dilated pharynx is associated with decreased pharyngeal contractility and resting tone and is a major risk factor for aspiration. The finding that some of the pharyngeal insult caused by the CPMD is permanent may support earlier intervention before end stage pharyngeal dilation occurs. This is similar to findings in esophageal achalasia, which support LES intervention before the development of an atonic, dilated, sigmoid esophagus [36, 37].

This investigation is not without limitations. Definitive conclusions cannot be drawn from this retrospective case series. As with all musculotendinous injuries, return to function and rehabilitation is generally measured in months rather than days or weeks [38], which exceeds the mean duration between the final dilation and fluoroscopic assessment of 37 days. Thus, it is possible that the maximum amount of improvement was not captured. In addition, we were not able to determine improvements between dilation procedures, so it is not known at what point patients experienced the greatest improvement. Our clinical experience suggests that a series of three double-balloon dilations with a gradual increase in balloon diameter provides the safest most effective treatment strategy. Previous investigation has reported lateral fluoroscopic PES opening improvement to 0.62 cm after single balloon dilation and 0.82 cm after myotomy [10]. While the improvement to 0.76 cm reported in this investigation suggests that a series of three sequential dilations provides improved outcomes over a traditional single balloon procedure, a randomized prospective comparison is required before definitive improvement can be confirmed. This may prove difficult, as dilation is often reserved for elderly persons with significant medical comorbidity and myotomy recommended for younger, healthier individuals. This study was limited to persons with complete survey (EAT-10) and fluoroscopic data after three procedures. Individuals who experienced significant improvement after one or two dilations were excluded, as were individuals who were lost to follow-up or declined a postoperative fluoroscopic swallow study. The influence of this follow-up bias has an uncertain effect on the improvement reported in this investigation.

This investigation was designed to determine the short-term symptomatic and objective outcomes of a series of three PES dilations. Previous investigations suggest there is inadequate long-term response following dilation to 20 mm, particularly among persons with CPMD secondary to neurodegenerative disease such as oculopharyngeal muscular dystrophy [7, 39]. Ideally serial dilation would provide longer duration of benefit. We do not have patients routinely return for reassessment unless their symptoms recur, but our clinical experience with this technique suggests some patients develop symptomatic recurrence with less severe degree of cricopharyngeus muscle obstruction that responds to a single repeat treatment. We reviewed our cohort and found there was a subset of patients who returned for additional treatments. In this relatively small series, 59% of patients needed treatment again at a later date, on average 416 days after the third dilation. This is congruent with reported rates and timing of recurrence of swallowing dysfunction following dilation of the cricopharyngeus, but the heterogeneity of the data included in these studies makes direct comparison of any individual techniques difficult [7, 12, 39]. Indeed, recurrence and durability are important considerations in the management of CPMD and appropriate patient counseling, so rigorous future prospective investigation to properly address this question are needed. Defining recurrence based on either symptoms or objective data is challenging and varies between patients, so our study advances understanding in what outcomes can be expected.

Though serial double-balloon dilation is performed at numerous centers, there is little literature on its safety. The key complication or side effect of concern in these procedures is upper esophageal perforation. During the study period we did not have any perforations of the upper aerodigestive tract. Presumably the risk of perforation is increased with greater dilation diameter. Mucosal lacerations occurred in some instances, but this was not discretely recorded and could not be measured. In our experience, a simple mucosal laceration did not lead to any instances of deep neck space infections or other complications. Although we believe progressive dilation over three serial encounters with appropriate recovery intervals reduces the likelihood of this complication, further investigation is required to confirm this assertion. Optimal interval between dilations is debatable, with repeat dilations for benign mucosal strictures of the esophagus often done in weekly intervals, but we believe CPMD treatment allows longer intervals between treatments without losing efficacy, given the apparent average duration of effect [12, 27, 28, 40]. Even with the inherent limitations of this retrospective case series, the data suggest that a “*series of three*” dilation approach is a safe and effective treatment of cricopharyngeus muscle dysfunction and support the need for further study.

## Conclusion

Our data suggest a “*series of three*” serial balloon dilation is a safe and effective treatment for CPMD. The treatment results in a significant improvement in symptomatic dysphagia (EAT-10), functional oral intake (FOIS), and objective fluoroscopic parameters. Further investigation is required to evaluate the durability and compare outcomes of this approach to traditional techniques of single balloon PES dilation.

## Abbreviations

CPMD: Cricopharyngeus muscle dysfunction
EAT-10: Eating Assessment Tool-10
FOIS: Functional Oral Intake Scale
PA: Pharyngeal area
PCR: Pharyngeal constriction ratio
PES: Pharyngoesophageal segment
PESAP: Pharyngoesophageal segment anteroposterior view
PESL: Pharyngoesophageal segment lateral view
SD: Standard deviation
VFSS: Videofluoroscopic swallowing study
